# Vaccine breakthrough infection and onward transmission of SARS-CoV-2 Beta (B.1.351) variant, Bavaria, Germany, February to March 2021

**DOI:** 10.2807/1560-7917.ES.2021.26.30.2100673

**Published:** 2021-07-29

**Authors:** Inge Kroidl, Ingo Mecklenburg, Peter Schneiderat, Katharina Müller, Philipp Girl, Roman Wölfel, Andreas Sing, Alexandra Dangel, Andreas Wieser, Michael Hoelscher

**Affiliations:** 1 Division of Infectious Diseases and Tropical Medicine, University Hospital, Ludwig-Maximilians-Universität (LMU) Munich, Germany; 2 German Center for Infection Research (DZIF), Partner Site, Munich, Germany; 3 Klinikum Landsberg am Lech, Internal Medicine, Landsberg am Lech, Germany; 4 Bundeswehr Institute of Microbiology, 80937 Munich, Germany; 5 Bavarian Health and Food Safety Authority (LGL), Oberschleissheim, Germany

**Keywords:** SARS-CoV-2, vaccination, breakthrough infection, transmission, B.1.351, unvaccinated partner

## Abstract

A breakthrough infection occurred in a fully Comirnaty (BNT162b2) vaccinated healthcare worker with high levels of neutralising antibodies with the SARS-CoV-2 B.1.351 (Beta) variant in February 2021. The infection was subsequently transmitted to their unvaccinated spouse. Sequencing revealed an identical virus in both spouses, with a match of all nine single nucleotide polymorphisms typical for B.1.351. To the best of our knowledge, no transmission of any variant of SARS-CoV-2 from a fully vaccinated person has been described before.

During a case cluster of severe acute respiratory syndrome coronavirus 2 (SARS-CoV-2) B.1.351 variant (Phylogenetic Assignment of Named Global Outbreak (Pango) lineage designation; World Health Organization designation Beta variant) [1,2] in a Bavarian hospital in Germany, in mid-February 2021, several transmissions to healthcare workers (HCWs) occurred. The first cases were diagnosed on 11 February and subsequent daily screening by rapid antigen test and twice weekly by RT-PCR of all staff and newly admitted patients identified several infected patients and 18 infected HCWs. Eight of the infected HCWs had already received one dose of Comirnaty (BNT162b2, BioNTech-Pfizer, Mainz, Germany/New York, United States) and one was fully vaccinated with two doses. No community transmission of the SARS-CoV-2 Beta variant outside the hospital was reported at that time.

Here we report the case of a breakthrough infection in a fully vaccinated HCW and the subsequent transmission of the virus to their spouse.

## Case description

The HCW in their early 60s was vaccinated with the first dose of Comirnaty in mid-January and received their second dose 21 days later, according to the recommendations at that time [3]. During regular screening the HCW tested negative, but became positive 26 days after their second vaccination. On the day of diagnosis, the HCW was free of symptoms and only experienced mild symptoms such as a headache and congested nose in the following days. Three days after the positive PCR of the HCW, their spouse developed influenza-like symptoms and was tested positive for SARS CoV-2 by RT-PCR. Starting with a sore throat, the spouse became more severely ill with fever and chills, followed by cough and dyspnoea for several weeks. Before the disease onset, the spouse who is working in a local service with customer contact but strict protective measures against coronavirus disease (COVID-19) in place, was working from home since 10 days because of a medical condition and did not have contact with friends or relatives in the 2 weeks before the infection.

## Laboratory investigations

In order to characterise the transmission chain, an extensive work-up of the cases’ samples was performed, as shown in the Figure. The RT-PCR test of the HCW was positive on 2, 5, 10 and 15 March, while for the spouse, several positive reverse transcriptase (RT)-PCRs were obtained between 5 and 17 March. Sequences were obtained from two swabs of the spouse taken on 5 and 17 March respectively, and a cell culture from swab on 5 March and from the HCW’s swab taken on 10 March.

**Figure:**
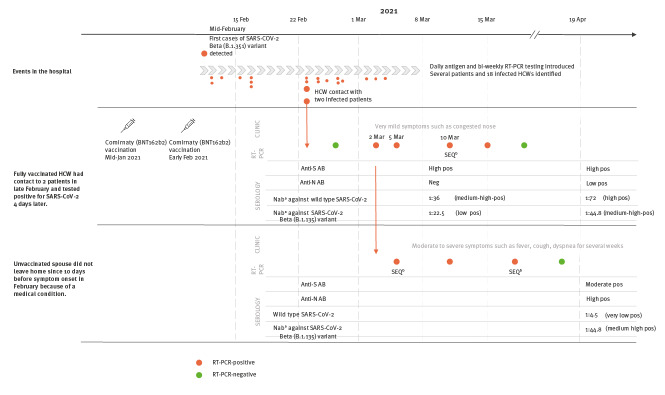
Timeline of events in a case of breakthrough infection in a fully vaccinated HCW and onward transmission of SARS-CoV-2 Beta (B.1.351) variant to unvaccinated spouse, Bavaria, Germany, February to March 2021

Anti-N AB: antibodies against anti-nucleocapsid protein; Anti-S AB: antibodies against anti-spike protein; HCW: healthcare worker; NAb: neutralising antibody; neg: negative; pos: positive; RT: reverse transcriptase; SARS-CoV-2: severe acute respiratory syndrome coronavirus 2; SEQ: sequencing; SNP: single nucleotide polymorphisms.

^a^ Corrected NAb titre from triplicates in cell culture.

^b^ SEQ and was performed and all nine defining SNPs for SARS-CoV-2 B.1.351 (Beta) variant (E:P71L, N:T205I, orf1a:K1655N, S:D80A, S:D215G, S:K417N, S:A701V, S:N501Y, S:E484K) were identical.

### Next generation sequencing

Swab samples or cell culture supernatants were used for viral RNA extraction and RT-PCR as described elsewhere [4]. SARS-COV-2 genome enrichment was performed after DNAse treatment with DNAse I and superscript IV (both ThermoFisher Scientific, Darmstadt, Germany) according to the manufacturer protocols with artic primers scheme V3 and Q5 polymerase (New England Biolabs, Frankfurt, Germany) according to [5]. Sequencing of SARS-CoV-2 enriched cDNA was performed after Illumina DNA prep on an Illumina Nextseq with 2 × 150pb PE. Bioinformatic analysis included bwa-mem mapping [6], ivar trimming and consensus generation [7] and pangolin software and nomenclature-based lineage classification [8,9].

Sequencing revealed that all nine defining single nucleotide polymorphisms (SNPs) for SARS-CoV-2 Beta (B.1.351) variant (E:P71L, N:T205I, orf1a:K1655N, S:D80A, S:D215G, S:K417N, S:A701V, S:N501Y, S:E484K) were identical (GISAID (https://www.gisaid.org/) sequence accession numbers: HCW (10.3.): EPI_ISL_3055531, spouse (17.3.): EPI_ISL_1640871).

### Neutralisation test

Neutralising antibody (NAb) titres were determined as previously described [10]. Briefly, SARS-CoV-2 (strain MUC IMB-1 and MUC-IMB-ZA1) was cultured in Vero E6 cells and NTs were performed in 96-well tissue culture plates (Greiner Bio-One, Frickenhausen, Germany) on confluent Vero E6 monolayers. Virus stocks (50 tissue culture infectious dose (TCID)/50 µl were prepared and stored at −80 °C until further use. Plasma samples (duplicates), including positive and negative control samples, were serially diluted in minimal essential medium (MEM), plus Non-Essential Amino Acids Solution and Antibiotic-Antimycotic Solution; all Invitrogen, ThermoFisher Scientific, Darmstadt, Germany) starting at 1:5 to a maximum of 1:640. Virus was pre-incubated with diluted plasma samples (1h, 37 °C) before the plasma-virus suspension was added to the cells. After 72 hours (37 °C) supernatants were discarded and wells were fixed (3% formalin/phosphate-buffered saline (PBS)) and stained (crystal violet, 0.1%). The NAbs titre corresponded to the reciprocal of the highest plasma dilution showing complete inhibition of CPE. A virus retitration was performed in triplicate on each plate and exact (corrected) titres were determined by retrograde calculation. The classification of titres into high, medium and low is based on observations of the results of numerous serum samples from SARS CoV-2-infected patients and vaccinated individuals (data not shown). Titres determined in virus neutralisation tests are highly method-dependent. The neutralisation assay we use here was developed primarily for screening suitable donors for convalescent plasma therapy. It is also used for the diagnostic identification of protective immunity in risk groups. The method was therefore optimised for the highest possible specificity and should ultimately be regarded as PRNT100. In other studies, however, PRNT50 tests are often used. These lead to apparently much higher titres in identical samples. In our hands, the typical titres we observe after basic/refresher vaccination range from 1:40 to 1:80. In contrast, the titres of naturally infected convalescent individuals a few weeks post-disease are typically 1:20 and below.

Serology from the HCW on day 7 after the first positive RT-PCR (9 March) revealed high titres against wild type-spike protein (Euroimmun Anti-SARS-CoV-2-ELISA anti-S1 (Euroimmun, Lübeck, Germany), optical density (OD) ratio 8.1 for IgG and > 9 for IgA, each positive > 1.1) and negative results for nucleocapsid antibodies (Roche Elecsys Anti-SARS-CoV-2 anti-N (Roche, Mannheim, Germany), cut-off index (COI) 0.12, cut-off for positivity (pos) > 1.0). Viral neutralisation showed an adjusted titre of 1:36 against wild type and 1:22.5 against B.1.351 SARS-CoV-2. A follow-up visit on day 40 (21 April) revealed increasing neutralisation titres of 1:72 against wild type SARS-CoV-2 and 1:44.8 against the B.1.351 variant. The HCW also seroconverted against nucleocapsid (COI 2.5; pos > 1.0).

For the spouse, the first available serology from day 37 after their first positive PCR (21 April) showed moderate/high titres against the spike protein (OD ratio: IgA 1.3, IgG 4.1; pos > 1.1) and high values for nucleocapsid antibodies, typical for unvaccinated convalescent individuals (COI 160.6; pos > 1.0). Sufficient neutralising capacity could only be demonstrated against the B.1.351 variant (1:44.8), but not wild-type SARS CoV-2 (1:4.5).

### Ethical statement

The described cases were enrolled into the Koco19 Immu-Study, approved by the Ethics Commission of the Faculty of Medicine at Ludwig-Maximilians-Universität (LMU) Munich, Germany under # 20-371.

## Discussion

Vaccination with Comirnaty has shown over 95% protective effect against SARS-CoV-2 infection and COVID-19 in large population studies of fully vaccinated individuals in Israel and elsewhere [3,11]. Full vaccination efficacy has been shown to be achieved starting 7 days after the second dose [12]. Breakthrough infections following transmission of SARS-CoV-2 have been described, however, mainly after only one dose of the vaccine was received by the index case [13]. To the best of our knowledge, no transmission from a fully vaccinated person has been described to date, as at 26 July 2021. In the cases described here, the index case (HCW) had high levels of anti-spike protein antibodies 33 days after their second vaccination and 7 days after the documentation of a SARS CoV-2 infection through a RT-PCR positive swab. Antibody levels in this range are seen after successfully completed vaccination with mRNA-based vaccines.

One limitation of our case report is that no blood samples are available from the early stages of the infection, hence it remains elusive if the HCW had a rapid boostering reaction, or if the measured antibodies were still present from the vaccination. Vetter et al. described that boostering during infection can occur quickly, i.e. as early as 4 days after symptom onset [14], whereas it usually takes at least 10 to 14 days to generate a measurable response. In our case, anti-nucleocapsid protein antibodies were negative at the study visit 7 days after the positive RT-PCR. This is consistent with an early course of a SARS-CoV-2 infection, as the vaccination with Comirnaty does not induce any response to the nucleocapsid protein and the HCW was naïve for that antigen. Vaccination with Comirnaty in the HCW was performed with a 21-day interval between the first and the second dose according to the respective initial recommendations [3]. Longer intervals of 4 weeks up to 6 weeks between the two doses were suggested later [15,16], which might lead to better protection against a SARS-CoV-2 infection.

## Conclusion

This breakthrough infection of a fully vaccinated HCW with onward transmission to an unvaccinated partner highlights the risk of transmission by fully vaccinated individuals to their close contacts. This might be especially applicable for individuals with high occupational risk for an infection, in outbreak situations and if working in hospital wards with acute COVID-19 cases.
